# Relationships between preadmission variables and academic outcomes for postbaccalaureate students in medical school

**DOI:** 10.1007/s10459-022-10129-3

**Published:** 2022-06-26

**Authors:** Stephen D. Schneid, Carolyn J. Kelly, Katharina Brandl

**Affiliations:** 1 Skaggs School of Pharmacy and Pharmaceutical Sciences, University of California San Diego, 9500 Gilman Drive (MC-0675), La Jolla, CA 92093-0675 USA; 2 School of Medicine, University of California San Diego, La Jolla, CA USA

**Keywords:** Admissions, Academic readiness, Diversity, Postbaccalaureate premedical programs, Preclerkship academic performance

## Abstract

There is currently little guidance for medical school admissions committees regarding how to weigh postbaccalaureate program grades relative to undergraduate grades. This study was designed to address this issue. Admissions data, preclerkship course performance and United States Medical Licensing Exam (USMLE) Step 1 results were analyzed over three years for University of California, San Diego (UCSD) postbaccalaureate premedical (PBPM) students (n = 25), students who participated in other postbaccalaureate programs (n = 34), and for the remainder of the medical students who did not participate in any postbaccalaureate programs (n = 329). UCSD PBPM program alumni did not significantly differ in their cumulative academic performance on exams in preclerkship courses and USMLE Step 1 pass rates compared to the rest of the class despite their significantly lower GPA, lower Biology, Chemistry, Physics and Math (BCPM) GPA, and Medical College Admissions Test (MCAT) percentiles. For students who participated in the PBPM programs, PBPM program GPA was a significant predictor of preclerkship academic performance and USMLE Step 1 performance. When assessing academic readiness of applicants who have completed postbaccalaureate programs, admissions committees might closely consider the postbaccalaureate program GPA in addition to other academic metrices such as BCPM GPA and MCAT score.

## Introduction

Postbaccalaureate premedical (PBPM) programs provide students an opportunity to demonstrate academic readiness on their medical school applications. This is most striking for students who have had academic difficulties in their undergraduate experience (Reeves et al., 2008). Most postbaccalaureate programs are designed to either help students change careers (“career-changer” programs) or to improve academic records (“academic-record-enhancer” programs) (Andriole & Jeffe, 2011). Career-changer PBPM programs are designed for students who have not completed premedical prerequisite coursework. These “start from scratch” programs have curricula that include a personalized learning plan and require courses such as Biology, General and Organic Chemistry, Physics, and Math. Students enrolling in this type of PBPM program do not necessarily have a weak undergraduate academic record. Academic-record-enhancer PBPM programs are designed to help students demonstrate an ability to perform well in upper-division science courses. Students enrolling in academic-record-enhancer PBPM programs generally have already taken the premedical prerequisites and some upper division science courses, but do not have a strong enough academic record to be a competitive applicant. There can be heterogeneity in terms of the specific upper division science courses and total units required in the academic record enhancer PBPM programs available. Both types of PBPM programs, especially academic record enhancer programs, open a pathway to medical school for some applicants whose admission based on undergraduate GPA metrics would be problematic for admissions committees. The number and percentage of medical school matriculants participating in such nondegree PBPM programs has increased from 1848 (12.7%) in 2009–2304 (14.5%) in 2021 (Andriole & Jeffe, 2011; Association of American Medical Colleges, 2021a, b). Additionally, the number of available PBPM programs has increased from 92 in 2009 to 226 in 2018 and 289 in 2021 (Association of American Medical Colleges, 2018, 2021a, b).

It is challenging to know how much weight to place on the postbaccalaureate program GPA when assessing academic readiness for medical school due to the significant variance in the structure and rigor of the programs. Even with a strong academic performance in a postbaccalaureate program, applicants may still have a lower overall GPA and MCAT score compared to other applicants (Andriole et al., 2015; Baill et al., 2019; Ganjoo et al., 2020; Giordani et al., 2001; Grumbach & Chen, 2006). Since admissions committees rely on these two academic metrics to provide insight about an applicant’s readiness for medical school in combination with the nonacademic factors used in holistic review, it is important to understand how postbaccalaureate program GPA as well as overall GPA or BCPM GPA, and MCAT scores relate to academic performance in medical school in this student cohort. There are limited studies examining medical school academic performance of PBPM students compared to non PBPM students. This is the first study to our knowledge that follows students from different types of PBPM programs to gain insight on the academic readiness of this group of students.

The theoretical basis of our study and research questions is rooted in the differential psychology literature that shows “the predictive validity of assessment procedures often improves when the predictor becomes more similar to the criterion” (Bergkamp et al., 2019). In a meta-analysis of eighty-five years of research on personnel selection methods, tests that require similar skills for success as the criterion were among the most valid predictors of future job performance (Schmidt & Hunter, 1998). We hypothesized that a medical student’s PBPM program GPA would be a strong predictor of academic performance if the PBPM program curriculum contained a rigorous course load of biomedical science courses that tested students on tasks that closely resemble those in medical school. This “curriculum sampling” approach to admissions has been studied in higher education (Niessen et al., 2018), but also specifically in medical education. For example, a study at a medical school in the Netherlands showed that selection of students who took an online course designed to mimic the courses and examinations in their medical program performed better than predicted by their pre-university GPA (pu-GPA), and performed as well as admitted students with high pu-GPA (de Visser et al., 2017). Using the applicant’s undergraduate GPA may not be as helpful in assessing academic readiness for medical school, especially for applicants from academic record enhancer programs. A study examining several years of student records from bachelor’s and master’s programs in computer science supports the approach of making admissions decisions that more strongly rely on grades earned in closer temporal proximity to the start of graduate programs (Zimmermann et al., 2017). Grades earned in closest proximity to the start of the graduate program significantly out-performed earlier grades when making correlations to the graduate GPA. Another study found that performance in a medical school summer prematriculation course taken just before the start of medical school correlated significantly with year 1 performance and was a strong predictor of year 1 performance (Schneid et al., 2018).

In this study, we sought to answer the following research questions: (1) How do the demographics and academic characteristics of medical students who have completed a PBPM program differ from medical students who have not completed a PBPM program? (2) How do medical students who have completed a PBPM program perform on preclerkship biomedical science coursework, and the United States Medical Licensing Examination (USMLE) Step 1 compared to students who have not taken a PBPM program? (3) How does the predictive value of PBPM program GPA for academic performance in the first two years of medical school and for USMLE Step 1 exam compare to other academic variables? Although recent research has studied GPA (Sladek et al., 2016) and MCAT scores (Agahi et al., 2018; Busche et al., 2020; Raman et al., 2019), our study's focus on the PBPM program GPA fills the gap in recent literature on the assessment of medical school applicants.

## Methods

### Context of this study

UC San Diego (UCSD) Extension, in partnership with the School of Medicine (SOM) and Division of Biological Sciences, launched an academic record enhancer PBPM program in 2013. The yearlong, 48-unit program is designed for first-time applicants and re-applicants to medical schools who have already completed their prerequisite coursework. Students take three courses during the summer, fall, winter, and spring quarters. In this program, students are required to take upper division courses in subjects foundational for medical school such as Physiology, Endocrinology, Pharmacology, Cell Biology, Immunology, Molecular Biology, Genetics, and Biochemistry through the UCSD Division of Biological Sciences. They also take advanced biomedical sciences courses with only their cohort in a team-based environment with frequent testing and feedback. Most students study for the MCAT and take the exam during the PBPM program. Every student is assigned one of the several PBPM program faculty advisors from the UCSD SOM to help guide them throughout the year. Students are also provided instructional support for MCAT preparation and given an opportunity to engage in a mock multiple mini interview. The program is designed to make students better learners and provide them with a strong biomedical science foundation for medical school. While UCSD PBPM Program graduates go on to attend medical schools across the United States, a significant proportion attend UCSD SOM. This continuity of location provides a unique opportunity to follow UCSD PBPM Program alumni from entry into the PBPM program through medical school.

### PBPM program types

Of the 388 UCSD medical students matriculating at UCSD SOM from 2015 to 2017, 25 students were graduates of the UCSD PBPM program. Thirty-four students participated in other non-UCSD PBPM programs (68% were “career changer” PBPM programs), and 329 students did not participate in any PBPM program.

### Students’ demographics

Demographic data from students matriculating at UCSD SOM from 2015 to 2017 were obtained from student records. Race or ethnicity was categorized as White, Asian, Underrepresented in Medicine (URiM) or Other. URiM students included African American, Hispanic or Latino, and American Indian or Alaska Native. The Other category included students that indicated mixed races unless one of the mixed race/ethnicity designations was URiM. In this case, students were classified as URiM. Gender, age, race/ethnicity, were compared between the graduates of the UCSD PBPM program, students who participated in other non-UCSD PBPM programs, and students who did not participate in any PBPM program.

### Prematriculation measures

Overall GPA (scale 1–4), Biology, Chemistry, Physics and Math (BCPM) GPA (scale 1–4), PBPM program GPA (scale 1–4), and Medical College Admission Test (MCAT) Total Score were compared between the three populations of medical students. For MCAT scores, the MCAT percentile ranks (scale 0–100) were compared, as some of the study cohort had taken the “old” MCAT (n = 225, scale 3–45), while the remainder took the “new” MCAT (n = 130, scale 472–528) launched in 2015. For students who took both the new and the old MCAT (n = 37), the new MCAT score was used for the analysis. Thirty-three students did not take the MCAT as they were accepted as part of a combined Bachelor’s-MD program. None of these students participated in a postbaccalaureate program. Undergraduate major was categorized into Natural Science, Social Science, or Other. The Other category included majors in Arts and Humanities as well as Business.

### Academic performance

Preclerkship performance (scale 0–100%) was calculated retrospectively for each student based on the average of student scores from the 20 course exams taken during the first and second year of medical school. The number of students who did not initially pass each first and second year course and required remediation was also assessed. USMLE Step 1 score (scale 1–300) and pass rate were compared between students who had completed the UCSD PBPM program, students who had completed a different PBPM program, and students who did not participate in any PBPM program.

### Statistical analysis

Statistical analysis was conducted using R statistical software (Team, 2014). Demographics and other characteristics were compared between three populations of medical students at the UCSD SOM: (1) Students who had completed the UCSD PBPM program, (2) students who had completed a different PBPM program, and (3) students who did not participate in any PBPM program. Chi-squared tests were used to compare gender, race/ethnicity, major, number of remediations, and USMLE Step 1 non-passing scores of the UCSD PBPM students, other PBPM programs, and students who did not take a PBPM program. Age at admission, GPA, BCPM GPA, MCAT percentile, USMLE Step 1 score were compared using one-way ANOVA with Tukey post-hoc test. Unpaired student’s t-test was used to compare differences in age across gender within each group. The following academic variables were used to predict preclerkship performance and USMLE scores: BCPM GPA, MCAT and PBPM program GPA. Relationships between various academic variables were calculated using Pearson’s correlation coefficients. Hierarchical multiple regression analysis was used to examine the contributions of each variable in the prediction of preclerkship performance as well as USMLE Step 1 performance. This allowed us to estimate the amount of variance in preclerkship performance and USMLE Step 1 performance. The type of prior PBPM program (UCSD or Other) was entered as a dummy variable and “no prior program” served as reference (Step 1). According to our hypothesis, we added next the academic variables based on their temporal proximity to the start of the graduate program. We started by adding the BCPM GPA (step 2) and then added MCAT next (step 3) which was taken during the PBPM program and in closer temporal proximity to the start of medical school. We used the BCPM GPA as preclerkship courses and USMLE Step 1 assess the application of important concepts of basic science to medical practice. The total GPA was not entered in the hierarchical regression analysis because the BCPM GPA constitutes part of the total GPA. Finally, a full model was examined (step 4) that included all four predictor variables: prior program, BCPM GPA, MCAT and PBPM GPA. Each single regression coefficient was tested for statistical significance using a t-test. To compare the different models the R^2^ change was tested with an F-test. Significance of the overall model was determined using an F-test. Multiple regression power analysis was performed using the pwr package in R (Champely, 2020).

## Results

### Demographics and academic characteristics

UCSD PBPM students, students who participated in other PBPM programs, and students who did not participate in any postbaccalaureate program were analyzed regarding their demographics and other academic characteristics. The three groups revealed statistically significant differences in race/ethnicity, age, GPA (total GPA and BCPM GPA), MCAT percentile and major (Table 1).

**Table 1:** Demographic and academic characteristics of UCSD PBPM students (UCSD PBPM program), students in other postbaccalaureate programs (Other PBPM program) or students who did not participate in any postbaccalaureate program (No PBPM program)

Characteristic	UCSD PBPM program	Other PBPM program	No PBPM program	p-value
Gender, n (%)				0.683
Male	10 (40)	16 (47)	161 (49)	
Female	15 (60)	18 (53)	168 (51)	
Race/ethnicity, n (%)				< 0.001
Asian	6 (24)	4 (12)	137 (42)	
White	11 (44)	13 (38)	139 (42)	
URiM	8 (32)	16 (47)	52 (16)	
Other	0	1 (3)	1 (0)	
Age at admission, mean (range)	24.8 (23–29)	28.0 (23–40)	23.1(19–33)	(a) < 0.0001
				(b) < 0.001
				(c) < 0.0001
GPA, mean (range)				
Total GPA	3.47 (3.14–3.91)	3.59 (3.12–3.98)	3.78 (3.13–4.00)	(a) 0.03
				(b) < 0.0001
				(c) < 0.0001
BCPM GPA	3.40 (2.98–3.90)	3.53 (2.92–3.99)	3.74 (3.01–4.00)	(a) 0.05
				(b) < 0.001
				(c) < 0.001
PBPM Program GPA	3.96 (3.73–4.00)	3.85 (3.51–4.00)	N/A	
MCAT Percentile, mean (range)				
Total MCAT	85.0 (54–99)	83.8 (58–99)	91.7 (51–100)	(a) 0.86
				(b) 0.002
				(c) < 0.0001
Major, n (%)				< 0.001
Natural Science	18 (72)	14 (41)	275 (84)	
Social Science	7 (28)	16 (47)	34 (10)	
Other	0 (0)	4 (12)	20 (6)	
Preclerkship Performance, mean (range)	82.6 (71.88–90.81)	82.0 (68.33–95.61)	84.9 (71.75–96.49)	(a) 0.92
				(b) 0.1
				(c) 0.01
USMLE Step 1, mean (range)	226 (203–251)	223 (155–259)	236 (177–272)	(a) 0.79
				(b) 0.02
				(c) < 0.001
USMLE Step 1 Nonpassing scores, n (%)	0 (0)	1 (3)	4 (1)	0.84
Remediations during preclerkship courses, n (%)	6 (24)	10 (29)	53 (16)	0.1

Chi-squared tests were used for categorical variables

One-Way ANOVA with Tukey post-test was used for continuous variables

*BCPM GPA* Biology, Chemistry, Physics and Math Grade point average, *GPA* Grade point average, *MCAT* Medical College Admission Test, *PBPM* Postbaccalaureate premedical, *USMLE* United States Medical Licensing Exam, *URiM* Underrepresented in Medicine

^a^Denotes comparison between “UCSD PBPM program” and “Other PBPM program”

^b^Denotes comparison between “UCSD PBPM program” and “No PBPM program”

^c^Denotes comparison between “Other PBPM program” and “No PBPM program”

URiM students accounted for 32% of the UCSD PBPM graduates, for 47% of students participating in other PBPM programs, and for 16% of the cohort who did not participate in any PBPM program. Students who had participated in any PBPM program were significantly older at admission. There was no significant age difference between males and females in any of the groups (data not shown). The two groups who had participated in PBPM programs both had significantly lower total GPA, BCPM GPA, and MCAT scores compared to the students who did not participate in any PBPM program. There was a significantly higher percentage of students with social science majors (28% for UCSD PBPM program graduates and 47% for other PBPM program graduates) compared to 10% for the remainder of medical students. No significant differences were found regarding gender.

### Performance and remediations in preclerkship courses

Despite the lower GPA and MCAT scores, UCSD PBPM program students’ exam performance in preclerkship courses was not significantly different from students who did not participate in a PBPM program. Students who participated in other PBPM programs, however, showed a lower performance compared to the rest of the class. No statistically significant differences were found between the groups for remediations during the preclerkship curriculum (Table 1).

### USMLE Step 1 performance

Both groups of PBPM program participants scored significantly lower on USMLE Step 1 exam compared to students who did not participate in any PBPM program. However, no significant differences were found regarding USMLE Step 1 passing rates between the three groups and none of the UCSD PBPM students failed the USMLE Step 1 exam on their first attempt (Table 1).

### Relationships between academic variables

Pearson correlation coefficients between variables are summarized in Table 2. Significant moderate positive correlations were observed between academic variables (Total GPA, BCPM GPA, PBPM Program GPA, and MCAT scores) and the two performance outcomes, preclerkship performance and USMLE Step 1 score. As BCPM GPA constitutes part of total GPA, a strong correlation of 0.95 was identified. The two performance outcomes, preclerkship performance, and USMLE Step 1 were also strongly correlated (r = 0.75).

**Table 2:** Pearson correlations among total GPA, BCPM GPA, PBPM Program GPA, MCAT Percentile, Preclerkship Performance and USMLE Step 1 score

Variable	1	2	3	4	5	6
1. Total GPA	–					
2. BCPM GPA	0.95***	–				
3. PBPM Program GPA	0.14	0.20	–			
4. MCAT Percentile	0.39***	0.40***	0.28*	–		
5. Preclerkship Performance	0.42***	0.41***	0.47***	0.40***	–	
6. USMLE Step 1	0.35***	0.35***	0.41**	0.46***	0.75***	–

*p < .05, **p < .01, ***p < .001

### Predictions of academic performance

Hierarchical regression analysis was conducted to evaluate the predictability of preclerkship score (Table 3). Hierarchical regression analysis revealed that the type of prior program contributed significantly to the regression model but accounted for only 3% of the variance in preclerkship performance. Introducing BCPM GPA (step 2) explained an additional 14% of the variance in preclerkship performance. In step 3, MCAT score was added to the analysis and the R^2^ changed 6% (step 3). Altogether, the three variables accounted for 23% of the variance in preclerkship performance. We replicated this hierarchical regression analysis for the cohort of all PBPM students. As step 1, we controlled for the type of prior PBPM program. Even though the type of prior program did not significantly correlate with preclerkship performance outcome, we elected to include this in step 1 of the regression analysis as this was part of our hypothesis. BCPM GPA explained 17% of the variance in preclerkship performance (step 2). The addition of the MCAT percentile accounted for an additional 10% of the variance. Beyond BCPM GPA and MCAT scores, the PBPM program GPA explained an additional 10% of the variance in preclerkship performance. All variables entered in the regression analysis accounted for 37% of the variance. Power analysis revealed that with 59 subjects and four predictor variables, a R^2^ as small as 0.16 can be detected with a power of 0.8 and a significance level of 0.05. Accordingly, we have sufficient power to detect the calculated adjusted R^2^ of 0.37.

**Table 3:** Hierarchical regression analysis for preclerkship medical school performance

Step	Variable	Beta	95% CI for B		SE	Std. Beta	Adjusted R^2^	∆R^2^
			LL	UL				
*Hierarchical regression analysis for all students (n = 329)*								
1	*Program*						0.03**	0.03
	No PBPM	Ref.						
	UCSD PBPM	− 2.32	− 4.55	− 0.09	1.14	− 0.10*		
	Other PBPM	− 2.87	− 4.81	− 0.93	0.99	− 0.15**		
2	*Program*						0.17***	0.14***
	No PBPM	Ref.						
	UCSD PBPM	0.90	− 1.30	3.10	1.12	0.04		
	Other PBPM	− 0.94	− 2.79	0.91	0.94	− 0.05		
	*BCPM GPA*	9.46	7.19	11.74	1.16	0.41***		
3	*Program*						0.23***	0.06***
	No PBPM	Ref.						
	UCSD PBPM	0.80	− 1.30	2.90	1.07	0.04		
	Other PBPM	− 0.52	− 2.30	1.26	0.90	− 0.03		
	*BCPM GPA*	7.07	4.67	9.46	1.22	0.32***		
	*MCAT*	0.15	0.10	0.21	0.03	0.27***		
*Hierarchical regression analysis for PBPM students (n = 59)*								
1	*Program*						− 0.02	− 0.02
	UCSD PBPM	Ref.						
	Other PBPM	− 0.55	− 3.72	2.61	1.58	− 0.05		
2	*Program*						0.17**	0.19***
	UCSD PBPM	Ref.						
	Other PBPM	− 1.84	− 4.77	1.10	1.47	− 0.15		
	*BCPM GPA*	9.44	4.37	14.51	2.53	0.46***		
3	*Program*						0.27***	0.10**
	UCSD PBPM	Ref.						
	Other PBPM	− 1.08	− 3.88	1.72	1.40	− 0.09		
	*BCPM GPA*	5.65	0.24	11.05	2.70	0.27*		
	*MCAT*	0.19	0.06	0.32	0.07	0.38**		
4	*Program*						0.37***	0.10**
	UCSD PBPM	Ref.						
	Other PBPM	0.74	− 2.13	3.61	1.43	0.06		
	*BCPM GPA*	3.91	− 1.25	9.08	2.58	0.19		
	*MCAT*	0.16	0.04	0.29	0.06	0.32*		
	*PBPM Program GPA*	15.74	5.41	26.07	5.15	0.37**		

*Beta* non-standardized regression coefficient, *BCPM GPA* Biology, Chemistry, Physics and Math Grade point average, *GPA* Grade point average, *LL* Lower Limit, *MCAT* Medical College Admission Test, *PBPM* Postbaccalaureate premedical, *SE* Standard Error, *Std. Beta* Standardized regression coefficient, *UL* Upper Limit, *USMLE* United States Medical Licensing Exam

*p < .05, **p < .01, ***p < .001

A similar analysis was conducted to predict USMLE Step 1 performance (Table 4). Hierarchical regression analysis revealed that the type of prior program contributed significantly to the regression model and accounted for 5% of the variance in USMLE performance. Introducing BCPM GPA (step 2) explained an additional 8% of the variance in USMLE performance. As step 3, MCAT score was added to the regression analysis and the R^2^ changed 11% (step 3). Altogether, the three variables accounted for 24% of the variance in USMLE Step 1 performance. Next, a similar hierarchical regression analysis was performed with the cohort of 59 PBPM students. After controlling for the type of prior PBPM program (step 1), BCPM GPA was added and accounted for 6% of the variance in USMLE performance. The addition of the MCAT percentile accounted for an additional 14% of the variance (step 3). In the final step, PBPM program GPA was added and the R^2^ changed 7%. All variables entered in the regression analysis accounted for 27% of the variance. Power analysis revealed that with 59 subjects and four predictor variables, a R^2^ as small as 0.16 can be detected with a power of 0.8 and a significance level of 0.05. Accordingly, we have sufficient power to detect our calculated adjusted R^2^ of 0.32.

**Table 4:** Hierarchical regression analysis for USMLE Step 1 performance

Step	Variable	Beta	95% CI for B		SE	Std. Beta	Adjusted R^2^	∆R^2^
			LL	UL				
*Hierarchical regression analysis for all students (n = 329)*								
1	*Program*						0.05***	0.05
	No PBPM	Ref.						
	UCSD PBPM	− 9.95	− 17.02	− 2.89	3.59	− 0.14**		
	Other PBPM	− 12.92	− 19.06	− 6.78	3.12	− 0.21***		
2	*Program*						0.13***	0.08***
	No PBPM							
	UCSD PBPM	− 2.28	− 9.51	4.95	3.68	− 0.03		
	Other PBPM	− 8.31	− 14.38	− 2.23	3.09	− 0.13**		
	*BCPM GPA*	22.54	15.06	30.02	3.80	0.31***		
3	*Program*						0.24***	0.11***
	No PBPM	Ref.						
	UCSD PBPM	− 1.10	− 7.90	5.69	3.46	− 0.02		
	Other PBPM	− 5.03	− 10.80	0.73	2.93	− 0.08		
	*BCPM GPA*	13.27	5.50	21.04	3.95	0.18**		
	*MCAT*	0.67	0.49	0.86	0.10	0.37***		
*Hierarchical regression analysis for PBPM students (n = 59)*								
1	*Program*						− 0.01	− 0.01
	UCSD PBPM	Ref.						
	Other PBPM	− 2.97	− 12.73	6.80	4.88	− 0.08		
2	*Program*						0.06	0.07*
	UCSD PBPM	Ref.						
	Other PBPM	− 5.62	− 15.29	4.05	4.83	− 0.15		
	*BCPM GPA*	19.54	2.86	36.22	8.33	0.31*		
3	*Program*						0.20**	0.14**
	UCSD PBPM	Ref.						
	Other PBPM	− 2.91	− 11.99	6.17	4.53	− 0.08		
	*BCPM GPA*	5.91	− 11.60	23.42	8.74	0.09		
	*MCAT*	0.69	0.27	1.11	0.21	0.44**		
4	*Program*						0.27***	0.07*
	UCSD PBPM	Ref.						
	Other PBPM	2.01	− 7.54	11.56	4.76	0.05		
	*BCPM GPA*	1.22	− 15.95	18.40	8.56	0.02		
	*MCAT*	0.61	0.21	1.02	0.20	0.39**		
	*PBPM Program GPA*	42.60	8.26	76.94	17.13	0.32*		

*Beta* non-standardized regression coefficient, *BCPM GPA* Biology, Chemistry, Physics and Math Grade point average, *GPA* Grade point average, *LL* Lower Limit, *MCAT* Medical College Admission Test, *PBPM* Postbaccalaureate premedical, *SE* Standard Error, *Std. Beta* Standardized regression coefficient, *UL* Upper Limit, *USMLE* United States Medical Licensing Exam

*p < .05, **p < .01, ***p < .001

## Discussion

With respect to our first research question regarding student demographics and academic characteristics, there was a relatively high percentage of the medical students coming from PBPM programs who were underrepresented in medicine, older, and majored in the social sciences. For example, in our study, 16% of medical students who did not take any PBPM program were underrepresented in medicine, a percentage comparable to the 21% of enrolled U.S. medical students in 2017 who were underrepresented based on race/ethnicity (Terregino et al., 2020). However, 32% of UCSD SOM students from the UCSD PBPM program and 47% from other PBPM programs were underrepresented in medicine. Increasing the number of medical students who are underrepresented in medicine helps create a physician workforce that is better reflective of the demographic characteristics of the US population (Lett et al., 2019). The benefits of having a physician workforce that better reflects the population include reinforcement of efforts to reduce health disparities and increase access to care for patients who are underserved by the healthcare system (Terregino et al., 2020). Furthermore, the greater trust patients have with physicians of the same race or ethnicity or who speak their native language results in better adherence to healthcare recommendations (Terregino et al., 2020). The additional life experiences that come along with being an “older” student also add to the diversity to the student body. Studying arts and humanities may help develop the competencies of professionalism, self-awareness, and communication skills that are central to the practice of medicine and deeply embedded within the core entrustable professional activities (EPAs) for entering residency (Chen et al., 2015).

With respect to our second research question regarding academic performance, students who completed the UCSD PBPM program had lower GPAs and MCAT percentiles, yet their performance on the medical school preclerkship course exams was similar to their classmates who did not participate in such programs. Students who completed other PBPM programs did perform lower compared to the rest of the class. The reason for this difference in preclerkship performance between PBPM programs could be that the UCSD PBPM program focused on foundational science courses for medical school such as physiology, endocrinology, pharmacology, and metabolic biochemistry. These courses prepared the students well for medical school coursework, whereas the majority of the other students participated in career-changer PBPM programs that focused on prerequisite coursework such as biology, chemistry, physics, and math. The percentage of students who participated in any postbaccalaureate programs and who required remediation of preclerkship coursework also was not significantly different from the rest of the medical school class. When combined with the observation that there was no significant difference in USMLE Step 1 pass rates, these data demonstrate that postbaccalaureate students progress as their classmates do through the preclerkship curriculum. While the USMLE Step 1 scores were lower for students from PBPM programs, this difference may not have an impact on competitiveness for residencies in the near future in light of the recent policy move to abandon the three-digit numeric score and implement a pass-fail reporting system (Pershing et al., 2020).

Students who participate in an academic record-enhancer type of postbaccalaureate program generally have an undergraduate GPA that raises concerns about academic readiness for medical school. For example, for the 25 UCSD PBPM alumni attending UCSD SOM, the average incoming BCPM GPA before starting the UCSD PBPM program was 3.16 (range 2.70–3.64), with seven students falling below 3.0. However, all these students did exceptionally well in the 48-unit UCSD PBPM program with an average program GPA of 3.96 (range 3.73–4.00). This program includes upper division biological science coursework on the UCSD main campus as well as advanced biomedical courses taken through UCSD Extension.

With respect to our third research question regarding the predictive value of the PBPM program GPA, our findings show that BCPM GPA, MCAT, and PBPM program GPA collectively accounted for 37% and 27% of the variances in preclerkship and USMLE Step 1 performance, respectively. Our results are in the expected pattern of magnitude that previous studies have demonstrated (Ferguson et al., 2002; Gauer et al., 2016; Kulatunga-Moruzi & Norman, 2002; Song et al., 2011). For example, in a recent study, the four new MCAT exam section scores explained 19% of the variance on USMLE Step 1 performance (Violato et al., 2020). Adding the PBPM program GPA explained an additional 10% and 7% for preclerkship and USMLE Step 1 performance, respectively. This small, but significant addition may be relevant for admissions committees that rely on BCPM GPA and MCAT scores, which are traditionally used by many medical schools (Albanese et al., 2003). Among the variables, MCAT and PBPM program GPA contributed significantly to the prediction of preclerkship performance and USMLE Step 1 scores, whereas BCPM GPA did not show a significant individual value of prediction when examined all together. Since most of the PBPM students take the MCAT during the program or afterwards, MCAT and PBPM Program GPA provide the most recent picture of students’ academic capability. This may illustrate the greater variance explained by the MCAT and PBPM Program GPA compared to the BCPM GPA.

The findings from a recent multi-site study showed that the MCAT total scores provided a prediction of academic performance in the first year of medical school for students from a broad range of sociodemographic backgrounds (Busche et al., 2020). In this study, correlations of MCAT total scores with academic performance in the first year of medical school ranged from “medium to large”, with a median correlation of 0.57. This is in concordance with our correlation analysis which revealed a medium correlation of MCAT scores as well as PBPM program GPA with preclerkship performance and USMLE Step 1 scores. Although MCAT scores and other preadmission variables have predictive validity and are a useful tool for admissions committees, other non-cognitive factors need to be considered for selection into medical school as well (Donnon et al., 2007).

The difference in the types of postbaccalaureate programs may be one reason for the differences in demographics and overall academic record between the students from UCSD PBPM program and other PBPM programs. For example, the UCSD PBPM program is an academic record enhancer type of program with curricular content and rigor that is very similar to the UCSD SOM curriculum. In addition, several UCSD PBPM students were accepted in each of the three years and it is possible that starting medical school with their classmates from the yearlong UCSD PBPM provided a support system to increase their academic success. For programs such as the UCSD PBPM program, students’ prior academic record is not reflective of their true potential, and we expected there to be a significant difference in pre-program versus program GPA (3.16 versus 3.96). On the other hand, the majority of the other postbaccalaureate programs were career changer type of programs with heterogeneous curricula that likely were not as focused on preparing students for medical school coursework as the UCSD PBPM program. Since career changer programs are generally aimed at having students take courses to fulfill the requirements for admission to medical school, students may already have had a strong academic record prior to entry into their program.

Our study’s results are similar to results found in the small number of previous studies examining the academic performance of postbaccalaureate program graduates in medical school. One recent study reported that students graduating from a “career changer” postbaccalaureate program had a “small but persistent score deficit” in eight of nine preclerkship course summative exams compared to students without postbaccalaureate experience (Baill et al., 2019). The average difference was 2.6% less (range 0.1–5.6%), which is nearly the same as the differences found in our study. The career changer students’ average USMLE Step 1 score was nine points less (228 versus 237). However, USMLE Step 2 CK scores did not significantly differ. Another study investigated performance of first-year medical students who had graduated from a postbaccalaureate program that combined biomedical content and research experience (Giordani et al., 2001). Similar to our findings with the UCSD PBPM students, this study found no significant difference in first-year medical school academic performance between students who had completed a formal PBPM program and those who did not, even though students who had completed a PBPM program had lower GPA and MCAT. They reported that preadmission variables such as GPA and MCAT could not predict academic success of the students from a formal PBPM program.

## Limitations

The main limitation this study was with respect to generalizability. There was a relatively small number of UCSD PBPM graduates in the three SOM classes we examined. Our study was also from a single institution and focused on a unique academic record enhancer type PBPM program, limiting the generalizability of our results. The numbers of UCSD PBPM graduates attending any other specific medical school was no more than a handful, limiting us to only examine UCSD SOM. However, we were able to identify students from other postbaccalaureate programs within the UCSD SOM classes besides the UCSD PBPM program and found similar results.

## Conclusion

The results of this study highlight that medical students who completed PBPM programs have similar preclerkship coursework performance and pass rates for the USMLE Step 1 as students who did not complete a PBPM program, despite having lower BCPM GPA and MCAT scores. Additionally, the more recent PBPM program GPA predicts future academic performance and thus academic readiness for medical school.
